# OMRT-6. Optimizing MDM2 inhibition for the treatment of high-grade glioma

**DOI:** 10.1093/noajnl/vdab070.031

**Published:** 2021-07-05

**Authors:** Veronica Rendo, Leslie Lupien, Nicholas Khuu, Kristine Pelton, Sophie Lu, Prasidda Khadka, Jaldeep Langhnoja, Timothy Phoenix, Keith Ligon, Pratiti Bandopadhayay, Rameen Beroukhim

**Affiliations:** 1 Dana-Farber Cancer Institute, Boston, MA, USA; 2 UC College of Pharmacy, Cincinnati, OH, USA

## Abstract

Over 80% of high-grade gliomas have alterations in members of the p53 pathway, a central regulator of cell cycle progression and apoptosis that becomes activated in response to cellular stress and DNA damage. For tumors that retain wild-type p53, pathway deregulation frequently occurs through the amplification of negative regulators of p53, including the E3 ubiquitin ligase MDM2. The p53/MDM2 interaction axis has served as basis for the development of several classes of MDM2 inhibitors, with AMG232 being the most potent molecule currently undergoing clinical evaluation. As the effects of MDM2 inhibition (MDM2i) remain poorly understood in high-grade glioma, we performed genomic and transcriptomic analyses in patient-derived models to better characterize sensitive tumors and identify putative biomarkers of drug response. Treatment with AMG232 impaired the growth of cell lines with wild-type p53 status, particularly in tumors with additional amplification of MDM4 or PPM1D activating mutations. Treatment with AMG232 upregulated both cell cycle arrest and apoptotic cellular responses, as measured by annexin V/PI staining and immunoblotting. Interestingly, the dynamics of these two downstream p53 signaling axis were dependent on treatment duration across models. In addition to p53 pathway activation and apoptotic induction, RNA-sequencing revealed MDM2i to be associated with the activation of oncogenic MAPK and KRAS signaling as well as epithelial to mesenchymal transition markers. In most solid tumors, resistance to MDM2i is mainly mediated by acquisition of p53 inactivating mutations. We hypothesized that resistance mechanisms in glioma may be partially driven by transcriptional changes, as these tumors consist of subpopulations with diverse cell differentiation states. By chronic AMG232 treatment, we have developed in vitro and in vivo models of acquired MDM2i resistance that are not mediated by p53 inactivation. Ongoing work is focused on characterizing the transcriptional profile of these cells to identify transcriptional changes leading to decreased drug response.

